# Angiogenesis in Regenerative Dentistry: Are We Far Enough for Therapy?

**DOI:** 10.3390/ijms22020929

**Published:** 2021-01-19

**Authors:** Oana Baru, Andreea Nutu, Cornelia Braicu, Cosmin Andrei Cismaru, Ioana Berindan-Neagoe, Smaranda Buduru, Mîndra Badea

**Affiliations:** 1 Department of Preventive Dentistry, Faculty of Dental Medicine, Iuliu Hatieganu University of Medicine and Pharmacy, 400083 Cluj-Napoca, Romania; oanabaru@gmail.com; 2 Research Center for Functional Genomics, Biomedicine and Translational Medicine, Iuliu Hatieganu University of Medicine and Pharmacy, 400015 Cluj-Napoca, Romania; andreeanutu.an@gmail.com (A.N.); braicucornelia@yahoo.com (C.B.); cismaru_andrei@yahoo.com (C.A.C.); ioana.neagoe@umfcluj.ro (I.B.-N.); 3 Stomestet Stomatology Clinic, Calea Manastur 68A Street, 400658 Cluj-Napoca, Romania; 4 Prosthetics and Dental Materials, Faculty of Dental Medicine, Iuliu Hatieganu University of Medicine and Pharmacy, Cluj-Napoca, 32 Clinicilor Street, 400006 Cluj-Napoca, Romania; 5 Department of Preventive Dentistry, Iuliu Hatieganu University of Medicine and Pharmacy, Avram Iancu 31, 400083 Cluj-Napoca, Romania; mebadea@umfcluj.ro

**Keywords:** angiogenesis, mesenchymal stem cells, dental stem cells, tissue regeneration, dental implants

## Abstract

Angiogenesis is a broad spread term of high interest in regenerative medicine and tissue engineering including the dental field. In the last two decades, researchers worldwide struggled to find the best ways to accelerate healing, stimulate soft, and hard tissue remodeling. Stem cells, growth factors, pathways, signals, receptors, genetics are just a few words that describe this area in medicine. Dental implants, bone and soft tissue regeneration using autologous grafts, or xenografts, allografts, their integration and acceptance rely on their material properties. However, the host response, through its vascularization, plays a significant role. The present paper aims to analyze and organize the latest information about the available dental stem cells, the types of growth factors with pro-angiogenic effect and the possible therapeutic effect of enhanced angiogenesis in regenerative dentistry.

## 1. Introduction

Angiogenesis is a process that takes place all along our lives, continuously, in presence of health, of injuries, of diseases. It refers to the formation of new blood vessels from the existing vasculature. According to Watt S et al., angiogenesis occurs in response to ischemia and hypoxia (events encountered post-surgically). Angiogenesis is characterized by extracellular matrix degradation and detachment of mural cells (pericytes or MSC-like cells) from capillaries and micro-vessels with a diameter of less than 100μm [1]. Through the newly formed vessels, oxygen, nutrients, various growth factors, immune system cells can contribute to better healing, and accelerate integration of a foreign body. Subsequently, angiogenesis is considered a critical factor in the success rate of guided bone and tissue regeneration in the dental field. It is fundamentally involved in bone grafts, barrier membranes, or dental implant materials [2].

It is essential to have a clear image of the angiogenesis process, of the cells involved, the cascade of factors that participate and control, the pathways they follow. Angiogenesis can either have a positive effect by being involved in wound healing or has also proved to have an active role in tumor growth and progression [3]. Oral-derived stem cells are able to secrete a wide range of angiogenic molecules that have signaling functions that spread biochemical signals, promoting cell renewal or cellular differentiation [4,5].

Angiogenesis is a complex multi-step process connecting extensive relationship between cells, soluble factors, and extracellular matrix (ECM) components [6]. These cells control the regional blood flow by releasing relaxing and contracting factors like nitric oxide (NO); metabolites of arachidonic acid that signals via cyclooxygenases, lipoxygenases, and cytochrome P450 pathways; peptides like endothelin, urotensin, C-type natriuretic peptide (CNP), and adrenomedullin; adenosine; purines; and reactive oxygen species [5]. The activation of the endothelial cells is linked to the signals received from the pro-angiogenic factors released by the surrounding cells. Following the injury, the EC secrete metalloproteinases (MMPs) responsible for disrupting of the basement membrane [7]. Vasodilatation is accompanied by extravasation of plasma that acts as a temporary scaffold for cell migration [8]. A proliferation of the EC occurs, and new blood vessels are formed, followed by a stabilization and remodeling of the new vessels by the pericytes. Also, a destabilization and a regression of unnecessary microvessels take place [8,9].

Endodontic angiogenesis is crucial to the long-term survival of regenerated pulp. The regeneration of pulp tissue rich in vascular-like structures is essential for restoring tooth vitality, process sustained by different injectable peptide scaffold wrapping the stem cell factor, that permit pulp regeneration rich in vascular-like structures [10].

Vascular endothelial cells are at the base of cellular-based therapy, drug discovery, as stated in a study published by Christensen et al., that reveals the differentiation of human pluripotent cells into endothelial ones, associated with TGFβ1-inhibitor, VEGF, and serum in the expansion medium; it results in a maintenance of the endothelial cell features and augmentation of their expansion rate [11].

It was observed an interplay among the dental cells and endothelial cells, affecting angiogenesis and pulp regeneration [12]. The entire angiogenesis process is mediated by a series of growth factors and mediators of the microenvironment components. One of the most significant factors is the VEGF—vascular endothelial growth factor family, with its receptors VEGFRs—vascular endothelial growth factor receptors [4]. Their prominent role resides in the EC’s activation, but also in skeletal development, in several stages on bone repair and regeneration [3,13]. There are multiple isoforms available VEGF-A, B, C, D, E and Placental Growth Factor (PLGF) [7,13]. VEGF-A is considered an essential member of this family, by being part of the blood vessel growth in the physiological pathological process [14]. For example, an excess of VEGF-A limits pericyte distribution by disrupting their migration along sprouting endothelial cells [13,14]. The VEGF-A is the prototype of all VEGFs. It has been proven that many viruses (oncogenic, infectious diseases) seek the upregulation of the host VEGF in order to disseminate their effect [14]. VEGF-B is involved in embryonic angiogenesis, VEGF-C and -D are part of lymphangiogenesis, and PlGF is an essential component of pathological angiogenesis [14].

The regenerative dentistry relies on the angiogenic process. Several stem cells available in the oral cavity were described along the years to improve soft and bone tissue regeneration. The present paper aims to systematize these cells to understand better the current literature and point out possibilities for accelerating the integration of dental implants and bone graft substitutes.

## 2. Dental Stem Cells—Their Proangiogenic Ability and Subsequent Regenerative Capacity

### 2.1. General Considerations

Stem cells are a population of undifferentiated cells that can widely proliferate (self-renewal) and differentiate into multiple types of cells (potent) [15]. It is vital to understand their biology, how they divide, replicate, or repair DNA [16]. Knowing their behavior is essential for developing their therapeutic potential, by using different signaling molecules empowering the immunosuppressive, immunomodulatory and regenerative potential of the dental stem cells.

According to their ability to differentiate or depending on their tissue origin, there are different types of stem cells. Based on their capacity to differentiate, there are two major categories: pluripotent and multipotent [17]. Pluripotent stem cells, also called embryonic stem cells (ESCs), can differentiate into every cell of the tissues derived from the three germ layers—endoderm, mesoderm, and ectoderm. In contrast, multipotent stem cells can differentiate into multiple cell types within one particular lineage [15,17,18]. The most known multipotent cell is the mesenchymal stem cells (MSCs). MSCs are described as promoters, enhancers, and playmakers in dental regenerative medicine [10]. The MSCs are able to differentiate into various tissues: adipose tissue, bone, cartilage, or muscles [19,20,21,22]. Besides the pluri and multipotent cells, based on the differentiation pattern, there also described the totipotent cells, encountered mostly during the embryogenesis period [23], the oligopotent cells capable of forming, two or more lineages of the same tissue, including—for example—the entire ocular surface of the pig [24], and the unipotent cells that can differentiate into only one specific cell type and form a single lineage [15].

The main two categories are the stem cells with the embryonic origin and the adult ones (somatic stem cells) based on their origin. In terms of wound healing, tissue repairing or regenerative medicine, these two categories raise up some issues: the ESC, although having a high proliferative capacity, they also imply legal and significant ethical dilemmas [25], the adult stem cells (ASC), found in the bone marrow and peripheral blood, are multipotent cells, limited in number and lineage-restricted [26]. The literature also describes the tissue-resident stem cells encountered in a “stem cell niche”, with a crucial role in stem cell homeostasis and tissue repair [27,28].

One particular category is the induced pluripotent cells (iPSCs), first described by Takahashi et al. and Yu et al., who were able to induce fibroblasts to gain pluripotency by identifying four essential transcriptional factors: Oct3/4, Sox2, c-Myc, and Klf4, or Oct4, Sox2, Nanog, and Lin28 [29,30]. These cells have numerous advantages compared to the ESC or adult stem cells, including easy harvesting from skin fibroblasts. However, any type of tissue can be a stem cell source: adipose, endothelial, dental. Being pluripotent, they can differentiate into any adult cell type, without triggering rejection [31,32]. The iPSCs are currently investigated in pre-clinical trials for three-dimensional (3D) organ printing, wound healing, angiogenesis [32] and have a great potential in cell-based therapy in bone and cartilage diseases [33,34,35]. There is a critical issue to be considered, that being the genetic stability of these iPSCs [33], that may generate tumor development in the targeted tissue [36]. In order to avoid tumorigenesis, a novel approach is being researched. The iPSCs derived extracellular vesicles are becoming a new strategy in regenerative medicine [36]. These exosomes (Exos) were identified as a subset of extracellular vesicles, participating in the intercellular communication in both physiological and pathological states [36,37,38]. Due to their capacity to induce and improve bone regeneration and angiogenesis, human iPSCs-MSC-Exos are also of high interest in dental regenerative field [38,39]. Nevertheless, before clinical use, the iPSC derived products need to be very cautiously screened because of the genetic risk alterations [40].

**Figure 1:**
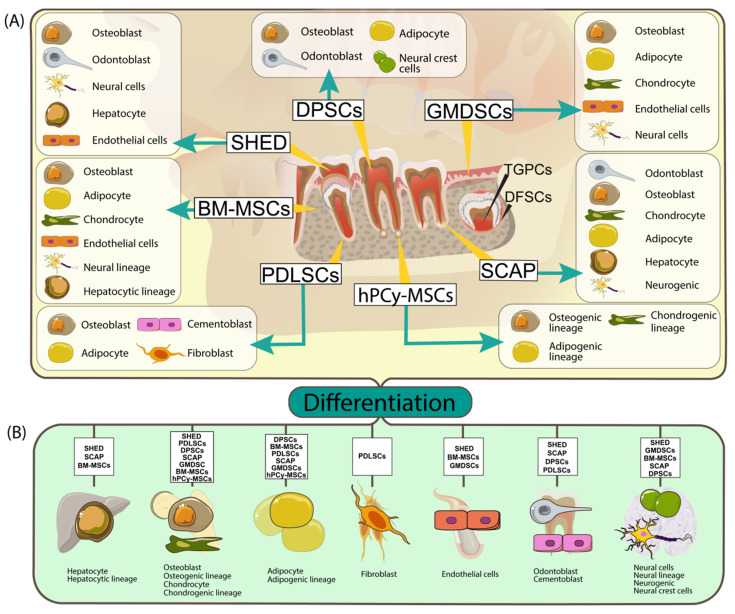
(**A**) Summary of dental stem cells types, sources and their multilineage differentiation capability (BM-MSCs: bone marrow mesenchymal stem cells; DPSCs: human dental pulp stem cells; SHED: human exfoliated deciduous teeth; PDLSCs: periodontal ligament stem cells; SCAP: stem cells from apical papilla; GMDSC: gingival-mesenchymal derived stem cells, hPCy- MSCs: human periapical cyst-mesenchymal stem cells). The yellow trace lines indicate stem cells origin and the arrows pinpoint their possible differentiation outcome; (**B**) Through specific environment conditions and different growth factors the dental stem cell types can be differentiated into other cell types (adipocytes, epithelial cells, hepatocyte, and so on).

Along the years, teeth and tissues present in the oral cavity have been described as an essential source of stem cells. Over the course of a lifetime, humans experience the growth of 20 deciduous teeth and 32 permanent ones. Every tooth has several components, significant for a better understanding of the MSc source. From the outside, the tooth crown is covered by enamel, underneath, we find the dentin, and deeper, the pulp, that hosts the nerve and blood vessels. The tooth root is covered by a cementum layer and communicates with the bone through the apical papilla. The entire structure is inserted in the alveolar bone. The two components are connected through different types of fibers that form the periodontal ligament. Above the bone, there is a soft tissue layer, represented by the gingiva. The components described above are illustrated in Figure 1. teeth are excellent tools for investigating organogenesis, angiogenesis, molecular biology, and homeostasis, due to their low morbidity and ease of access.

Summarization of dental stem cells types, emphasis their specific differentiation line in function of the specific subtype (BM-MSCs: bone marrow mesenchymal stem cells; DPSCs: human dental pulp stem cells; SHED: human exfoliated deciduous teeth; PDLSCs: periodontal ligament stem cells; SCAP: stem cells from apical papilla; GMDSC: gingival-mesenchymal derived stem cells).

### 2.2. Bone Marrow Mesenchymal Stem Cells (BM-MSCs)

The MSCs collected from the bone marrow are probably the most intensively studied types of stem cells. However, those of dental origin are at a more incipient level [39]. Besides the source, the study of the MSCs relies on the knowledge of the differentiation process, signaling pathways, transcription factors and molecules that control and influence the entire cascade, starting from the isolation point and going through the inflammatory and immune response. BM-MSCs have proven challenging to isolate due to the high presence of hematopoietic cells and their capacity to differentiate when lacking differentiation culture conditions [40]. The International Society for Cellular Therapy (ISCT) defines mesenchymal stem cells by multipotency, the capability of adherence to plastic and the positive and negative expression of a well-established panel of surface antigens (Table 1) [41]. Over the course of the last years, STRO-1, CD271/NGFR, CD200, Ganglioside GD2, CD348 MSCA1, and others have been introduced as markers to verify MSC identity with markers differing between tissue type (e.g., bone marrow, dental pulp, adipose tissue, peripheral blood) and between species (e.g., humans, mice) [42].

Their immunomodulatory function can be evaluated by suppressing the effector leukocytes, like T lymphocytes or monocytes [43]. The immune reaction developed by the MSCs can either be due to their direct contact with immune cells or to the secretion of reacting molecules according to the host tissue [44]. This means that depending on the pro-inflammatory and anti-inflammatory proportion of cytokines at the site of the surgery, the MSCs can be responsible for the transforming growth factor beta (TGF-β) expression that induces several of immune regulatory cells, like antigen-presenting cells (APCs) or natural killer cells (NKCs) [45]. The immunomodulatory activity of the MSCs has been studied in clinical situations, such as graft versus host disease, Crohn disease, type 1 diabetes, acute pancreatitis multiple sclerosis [46,47].

Bone healing depends on angiogenesis. In a study conducted by Takeuchi, it is proven that the human bone marrow mesenchymal stem cells with their components, the exosomes, including the miRNAs—such as miR-196a, miR-27a, and miR-206—were involved in the angiogenesis and bone repair process, at an early stage, by activating the VEGF family factors and osteogenesis paracrine factors in the receptor cells [43].

A paper published by Mashimo in 2019 presents an experimental study on mice, where the upper first molar was extracted and immediately after BM-MSCs (PDGFRα+, Sca-1+, CD45−, TER119—cells) from the tibia and femoral were immediately isolated and applied into to extraction sockets. After six weeks, they have proven an accelerated bone healing with an increased quantity of bone marrow [44].

### 2.3. Human Periapical Cyst-Mesenchymal Stem Cells (hPCy-MSCs)

The human Periapical cyst mesenchymal stem cells- hPcy-MSCs was described as a subdivision of this category, a specific type of cells with origin in the dental periapical cyst [48]. Endodontic infections due to improper dental treatment, anatomical impairments, insufficient knowledge or equipment, may lead to development of inflammatory periodontitis, a fibrous inflammatory tissue, with abundant number of macrophages, lymphocytes and neutrophils [49]. The periapical granulation tissue may undergo a cystic transformation if a sufficient amount of time passes by [50].

hPCy-MSCs were firstly described by Marrelli et al. [51], isolated from human periapical cysts. The hPCy-MSCs were indicated to have self-renewal capacity and multi-lineage differentiation potency, which permit their differentiation into osteoblasts, adipocytes and chondrogenic lineage. Freshly isolated hPCy-MSCs express similar markers with other type dental-derived MSCs, highly express CD13, CD29, CD44, CD73, CD90, CD105, STRO-1, and CD146, and do not express hematopoietic markers, such as CD45 [49]. They do express the main neuronal markers, such as β-III tubulin, and the main astrocytes markers, such as glial fibrillary acidic protein (GFAP) [52] and higher expression of transcripts for neuronal markers (β-III tubulin, NF-M, MAP2) and neural-related transcription factors (MSX-1, Foxa2, En-1) as compared with dental pulp stem cells [53]. Other markers expressed by these cells are: CD13, CD29, CD44, CD73, CD90, CD105, STRO-1, and CD146 [49]. This last marker, CD146, was investigated in order to establish its role in regulating the osteogenic differentiation of the hPCy-MSCs. The CD146 Low population proved a significantly higher osteogenic differentiation capacity with respect to the CD146 High counterpart, meaning that CD146 has a major role in regulating stem cell properties of hPCy-MSCs [54].

One major advantage of this cell population is the easy way to be harvested, periapical cysts being considered a biological waste. They are to be surgically removed, with no influence on the healthy oral tissues. Being widely spread and with a cell surface marker profile similar to that of other oral derived MSCs, they represent an alternative to be considered in regenerative medicine [55].

#### Potential Clinical Approaches of HPcy-MSCs

hPCy-MSCs might be a useful source of MSCs with therapeutic properties for regenerative medicine and tissue engineering [51]. This cells with MSCs features permit their differentiation in cystic fibrous tissue under continuous, in the case of untreated pulpo-periodontal infection, having important role in regenerative medicine [49]. However, the most important features is related to the recently discovered neural plasticity, that undoubtedly constitutes a standpoint aspect to be additionally explored for prospective innovative strategies in brain repairing [49]. The application of this type of MCS cells can be developed after the deciphering of the immunodulatory features, as found in a limited number of in vitro studies.

The hPCy-MSCs-derived exosomes released in the culture medium of dopaminergic neurons may offer a strategy aimed to improve the early diagnosis and to find novel therapeutic strategies for Parkinsons’ disease [56]. In terms of osteogenesis, hPCy–MSCs showed increased mRNA levels of bone specific genes, such as osteocalcin (OSC), osteopontin (OPN), alkaline phosphatase (ALP), and dentin matrixprotein 1 (DMP-1), after osteogenic induction, proving that they are more oriented towards osteogenesis than DPSCs, which are more likely directed toward dentinogenesis [55].

### 2.4. Human Dental Pulp Stem Cells (DPSCs)

The dental pulp is soft connective tissue inside the crown and root of a tooth, hosting the blood vessels and nerves inside the root canal through a foramen, called the apical papilla. The cells found inside the pulp chamber are endothelial cells, fibroblasts, osteoblasts, osteoclasts, and odontoblasts [45]. Being a tissue with a mesenchymal origin, multipotent, that expresses mesenchymal markers like CD29, CD44, CD59, CD73, CD90, CD146, can differentiate in multi-lineage [46]. Compared to the BM-MSCs, the DPSCs have a higher proliferation rate [46]. Potential use of DPSCs appears documented in fields like: neurological diseases [57]; in ischemia: due to pro-angiogenic factors like VEGF-A, G-CSF, GM-CSF, and MMP3, they are able to improve the angiogenesis [58]; in Duchenne muscular dystrophy by promoting angiogenesis and by diminishing fibrosis [59]; in peripheral nerve injury, due to their ability to produce neutrophil factors such as: BDNF, NGF, NT-3, and GDNF, responsible for nerve regeneration or by an affinity to glial differentiation [60]; in bone reconstruction, as suggested by studies conducted by Maraldi et al., where the hDPSCs and amniotic fluid stem cells (AFSCs), combined on a collagen scaffold, reconstruct large-size bone defect on animal model; in liver diseases, having anti-fibrotic and anti-inflammatory properties [60]; in myocardial infarction by reducing the infarct size, improved angiogenesis and wall thickening of the heart left ventricle anterior wall, as proven in a study conducted on rats [61]; in eye diseases, the studies were more promising in vitro, than in vivo, where they failed to differentiate into neuron and integrate into the retina [62]; due to the neurotrophic factors production, such as the nerve growth factor, glial-cell derived neurotrophic factor, brain-derived neurotrophic factor, bone morphogenetic protein 2, research is also oriented towards neurodegenerative diseases, such as Alzheimer’s [63].

A significant advantage of these type of cells is their potential source. For example, teeth are frequently extracted for orthodontic purposes, and the DPSCs collected can be cryopreserved until needed and even expanded. One proven use is improving diabetic polyneuropathy through their ability to increase nerve blood function and nerve conduction [64].

Since their source is located inside the oral cavity, it came only naturally to conduct studies to treat various conditions and diseases. The application of hDPSCs in regenerative endodontics procedures has been widely studied. In pulp inflammations, pulpitis, the activation of complement C3a receptor, is present on the hDPSCs and dental fibroblasts, which improves the hDPSCs proliferation and dental fibroblast recruitment [65]. The DPSCs have superior qualities than BM-MSCs in situations with local inflammation due to their downregulated mitogen-activated protein kinase (MAPK1) gene expression, involved in the ROS (reactive oxygen species) level and subsequently in the senescence process of cells [66]. Many studies focused their attention on the liaison between periodontitis and hDPSCs. For example, the association between the hepatocyte growth factor (HGF) and the hDPSCs improved periodontal bone regeneration in swine [67], in a study performed on dogs, with canine periodontitis model, the researchers evaluated the ability of DPSCs to regenerate periodontal defects, proving significant results in cementum and periodontal ligament formation where DPSCs were associated with xenograft materials (bovine mineral graft—Bio-Oss) into the periodontal pokes [68]; other study conducted on miniature pigs, focused their attention on regenerating periodontal defects by associating hDPSCs from inflammatory pulp tissue with β-tricalcium phosphate (β-TCP), obtaining significant results in terms of new attachment formation [69].

Bone formation and hDPSCs had to be also investigated. Orthognathic surgery relies on bone healing with a significant impact in function and aesthetics. Distraction osteogenesis is one of the maneuvers, and a study of rabbits proved that the association of Sirtuin 1 (SIRT1), a nicotinamide adenine dinucleotide (NAD), and hDPSCs ameliorates osteogenic differentiation and subsequently bone regeneration [70].

A recent study, analyses the DPSCs combination with various growth factors and scaffolds, proving than a right, proper combination could lead to an improved, more efficient DPSCs properties [71].

### 2.5. Human Exfoliated Deciduous Teeth (SHED)

Stem cells from the human exfoliated deciduous teeth are another significant source of regenerative potential in medicine, presenting the advantage of easy harvesting and low morbidity in surgical procedures. Compared with other oral sources of stem cells, like DPSCs or periodontal ligament stem cells (PDLSCs), SHED experienced a higher rate of proliferation [72], sustained by a positive expression of CD71 (marker present in proliferating cells) and CD105 [72] (a marker found in endothelial cells) [73] and a higher expression of genes encoding basic fibroblast growth factor (bFGF) and bone morphogenetic protein 2 (BMP2). The bFGF is involved in the angiogenesis, bone and cartilage formation process, and the BMP2 in the osteoblast differentiation, making the SHEDs ideal candidates in bone regeneration therapy [74,75]. SHED has an advantage: being younger and immature cells, they are more likely to promote regeneration in various tissues, accelerating angiogenesis and potential to differentiate into sensory neurons and odontoblasts [75].

Similar to DPSCs, the dental pulp from exfoliated deciduous teeth can be cryopreserved. Subsequently, stem cells can be obtained, proving promising results in mineralized tissue around the implants site, in addition to hydroxyapatite tricalcium phosphate (HA/TCP). The data of the study shows that SHEDs-Cryo (cryopreserved) have unique regeneration properties, the cells’ biological and immunological qualities are similar to the freshly isolated SHED (SHED-Fresh) [76].

SHED has also been studied through its immunomodulatory effects, showing properties in reducing inflammation, reducing apoptosis, maintaining a normal salivary flow, effects studied on mice, an animal model with Sjögren syndrome (an autoimmune disease) [77]. SHEDs proved abilities in dentine-pulp regeneration, bio-root, and periodontal regeneration [78].

### 2.6. Periodontal Ligament Stem Cells (PDLSCs)

The periodontal ligament (PDL) is the connective tissue responsible for the attachment between the tooth root covered by a mineralized tissue, the cementum, and the alveolar bone. Its primary role is to support the tooth, but it also ensures the nutrition and the repair of various local lesions [79]. Apart from fibers, the periodontal ligament also contains nerves and blood vessels, responsible for fine proprioception. Like other stem cells with a buccal origin, these cells, can also be obtained and isolated from teeth extracted for orthodontic reasons, impacted third molar, or supra-numerous teeth, and advanced mobility with irreversible periodontitis, with fewer concerns about ethical problems [80].

Periodontitis is a multifactorial disease, an infectious disease, with many types of bacteria involved in the destruction of the periodontal ligament, and the cementum of the tooth, the alveolar bone, leading to tooth mobility and loss. Since it is widespread, in 2004, a group of researchers focuses their attention on proving that the periodontal ligament contains stem cells that could be used to repair lost, damaged periodontal tissue, being able to regenerate cementum and PDL-like tissue in immunocompromised rats [81]. This regenerative potential of the PDLSCs remains unaltered even in cases of inflammation of the periodontium. The PDLSCs isolated from inflammation tissue, were able to regenerate cementum like structures and PDL fibers [82]. While inflammation does not affect their potential, the donor‘s age, on the other hand, seems to have a negative impact in terms of PDLSCs cell number, proliferation, and differentiation potential [83].

Researchers have tried to find along the years various ways to optimize the periodontal regeneration. One method that showed promising results is the use of PDLSCs seeded on a biphasic calcium phosphate scaffold (BCP), obtaining new bone formation [84]. Another study that used 3D printing polycaprolactone (PCL) scaffold realized growth factors like: connective tissue growth factor (CTGF) and bone morphogenetic protein 2 and 7 (BMP-2, BMP-7) and PDLSCs added. This data sustains the hypothesis of potential cementum formation on root tooth surfaces [85].

### 2.7. Stem Cells from Apical Papilla (SCAP)

Among the sources of stem cells with differentiation potential found in the dental tissues, the apical papilla has been isolated and characterized. It was only in 2008 that Sonoyama et al. published a paper proving the existence of a new population of stem cells that were isolated from the root apical papilla of human teeth [86]. The SCAP were identified due to their positive response to STRO-1, an early progenitor mesenchymal marker and the expression of CD146 [87].

In a comparison with other populations of stem cells. SCAPs proved higher proliferation rate, great tissue regeneration abilities and improved migration capacity in a scratch essay, compared to the DPLSCs [86,87], properties that remained at a higher level even when compared to the PDLSCs, the SCAPs showing better mineralization capacity than the PDLSCs [88,89]. A series of molecular markers have been used to distinguished SCAPs from other stem cells of oral origin. In this regard, the CD24 marker is undetectable in the DPSCs, although both are positive for STRO-1, CD146, and CD34 markers [82,85]. The SCAPs properties in terms of proliferation and mineralization remains higher even in comparison to the DPSCs [88,89].

SCAPs are an interesting source of cells also due to their multi-lineage differentiation, being able to differentiate into odontoblasts and osteoblasts [90]. Their neurogenic differentiation was also proven in vitro [91]. Neural differentiation of SCAPs might be enhanced by the use of graphene dispersion (GD) and water-soluble single walled carbon nanotubes (ws-SWCNT) [92]. SCAPSs also present the capacity to differentiate into adipocytes, chondrocytes, or hepatocytes [93].

SCAPs represent a unique source of stem cells, of large interest and great therapeutic potential. Their high proliferation rate, low immunogenicity, and cryopreservation features, make them an alternative to be considered in the stem cell therapy [94]. The angiogenic property of SCAPs was proven both in vivo and in vitro, by promoting migration and tube formation of existing vascular cells [95] and expressing higher levels of angiogenesis-related genes [96]. The SCAPs involvement in the angiogenic process is also sustained by their ability to secrete proangiogenic molecules, like angiogenin, VEGF and insulin-like growth factor binding protein, that enhance the angiogenic potential of endothelial cells, directly responsible for the angiogenic reparatory scheme [97,98]. Nevertheless, the proangiogenic effect of SCAPs under hypoxic environments is increased [95,98].

The SCAPs therapeutic potential has been studied in terms of endodontics, bio-root engineering, periodontal regeneration and bone repair [99]. Multiple types of scaffolds, starting with the hydroxyapatite/tricalcium phosphate (HA/TCP) [84,99], continuing with decellularized dental pulp [99] and scaffold-free stem cell sheet-derived pellet (CSDP) [100] have been developed in order to create a conductive environment for odontogenic differentiation and dentin pulp regeneration.

### 2.8. Gingival-Mesenchymal Derived Stem Cells (GMDSC)

One of the periodontium components is the gingiva, next to the periodontal ligament, root cementum and alveolar bone. The gingiva is the most superficial and therefore accessible of all the layers. It is commonly undergoing surgical interventions during tooth extractions, periodontal interventions, gingivectomy or dental crown lengthening, with a remarkable ability to repair small scars or any scars at all [101,102]. From a histological point of view, the gingiva is composed of stratified squamous epithelial tissue supported by a dense fibrous connective tissue stroma termed lamina propria [103]. In terms of specific mesenchymal markers, the GMDSCs presented in flow cytometry analysis CD44, CD73, CD90, CD105, including STRO-1, CD146, CD166, and CD271, but did not express hematopoietic antigens [41,104]. The CD105, CD73, and CD90 are considered essential criteria for the MSCs [41,104].

Similar to other MSCs from the oral cavity, the GMSCs present a multi lineage differentiation potential in osteoblastic, adipocytic, chondrocytic, endothelial, and neural directions [105,106,107]. Regarding the correlation between the angiogenic process and GMDSCs, a recent study, published in 2020, demonstrated that the over expression of the fibroblast growth factor 2 (FGF-2) promotes the GMSCs role in the blood vessel formation [108].

### 2.9. Dental Follicle Stem Cells DFSCs

The tooth development and eruption is directly connected and mediated by the human dental follicle, which is a connective tissue sac derived from ectomesenchymal tissues, part of the tooth germ [109]. The dental follicle cells have origin in the neural crest, playing an essential role in development of the periodontium and activation of the osteoclasts, necessary for bone resorption during teeth eruption [110]. These particular stem cells, isolated from the dental follicle of impacted third molars, were first described and characterized by Morsczeck et al. in 2005 [111]. Over the years, the DFSCs caught the attention of researchers for many reasons. Their multi potential to differentiate into cementoblasts, chondrocytes, adipocytes, and osteoblasts is sustained by their content of heterogeneous populations of stem cells, which are derived from mesoderm and ectoderm: embryonic stem cells, mesenchymal, neural progenitor cells, and neural crestal stem cells [112]. The DFSCs are able to differentiate into neural-like cells, when cultured in a neural inductor medium [113]. These cells were proven to possess characteristics of mesenchymal stem cells (MSCs) including self-renewing capacity, plastic adherence, expression of specific surface markers: CD73, CD90, CD105 [114]. hDFSCs have been shown to secrete a significant number of essential factors, such as matrix metalloproteinases (MMPs), insulin-like growth factor (IGF), vascular endothelial growth factor (VEGF), basic fibroblast growth factor (bFGF), and hepatocyte growth factor (HGF), factors with a key role in angiogenesis, immunomodulation, and regenerative process [114].

One important aspect is that these cells, similar to the hPcy-MSCs, are considered a biological waste. Frequently, wisdom teeth are removed for orthodontic purposes, subsequently, the dental follicle sac as well [109]. This offers the researchers a great amount of material in order to investigate various directions in therapy. A significant part to be taken into consideration, is the senescence, that was proven to affect the use of DFCs in stem cell therapies and needs to be regulated [115].

#### Potential Clinical Approaches of the DFSCs

Similar to other cells, harvested from the oral cavity, DFSCs were included in the tissue engineering research. DFSCs are progenitors of alveolar osteoblasts, subsequently a new source for bone tissue engineering [116]. DFSCs are able to differentiate into osteoblast-like cells, producing mineralized matrix nodules and expressed the typical osteoblastic markers, Alkaline Phosphatase (ALP) and Collagen I (Coll I) [117]. This osteogenic potential must be carefully manipulated, as while β-tricalcium phosphate supports the osteogenic differentiation in DFCs it also induces apoptosis [118]. Regarding the neural potential, DFSCs can successfully differentiate into neural like cells, but compared to the SHED, they have different neural differentiation potential under the same medium conditions [119]. Not only is the bone regeneration sustained by the DFSCs, but also the periodontal one. The role of DFSCs was demonstrated in patients diagnosed with periodontitis, where the affected PDLSCs enhance the activity of DFSCs in producing more anti-inflammatory cytokines and trophic factors [120].

## 3. Experimental Therapeutic Applications of Oral MSCs in Oral Diseases

For the inflammatory diseases, such as the periodontal disease, and various infections in the oral cavity, it was only natural to start the search for possible therapeutic solutions with the oral MSCs, considering their multi-lineage potential (Table 2).

### 3.1. Endodontic Diseases

One of the most common disease of the oral cavity is dental caries, which may lead to the lesion of the pulp-dentin complex. The dental pulp occupies the central chamber of the tooth crown, continuing its way through the tooth root. This complex is defined by two networks: nerve fibers and blood vessels [122]. The bacteria from an untreated carie may advance its way to the dental pulp, causing pulpitis or necrosis. Besides the traditional treatment, with filling and root canal treatment, especially in immature teeth, where the apical papilla and the apex is still open, regenerative endodontics has been increasingly studied.

Two types of MSCs have been proven with more advantages in regenerative endodontics, DPSCs and SHED. These two types of cells proved their quality in inducing neovascularization with complete, functional pulp regeneration [122]. Depending on the clinical diagnosis, there can be two treatment directions, either partial dental-pulp regeneration, or entire dentin-pulp regeneration [122]. Tissue engineering is based on combined stem cells, growth factors, scaffolds, exosomes [123,124,125,126].

Three-dimensional (3D) scaffolds are frequently used in tissue engineering to support cell growth. Since there are concerns about the complete degradation or remaining products that may impair tissue regeneration, scaffold-free 3D constructs, could be the answer [127]. Authors Itoh and Sasaki, proved that 3D DPSC scaffold free construct can be successfully used in obtaining dental-pulp regeneration, blood vessel–rich pulp-like tissues could be formed with DPSCs without requiring scaffolds and growth factors [127].

Another direction of potential regeneration could be the enhancement of DPSCs properties by combining growth factors with angiogenic, proliferative and odontogenic abilities. Platelet-derived growth factor (PDGF) was originally identified in platelets, and there are five polypeptides included in the family: PDGF-AA, PDGF-AB, PDGF-BB, PDGF-CC, and PDGF-DD [128]. One of them, PDGF-BB, has indirect angiogenic effect through its promotion of vascular endothelial growth factor (VEGF) secretion, playing an important role in maintaining the stabilization of newly formed blood vessels [126]. hDPSCs can secrete VEGF which are necessary for complete pulp healing [65]. Zhang et al. proved that the overexpression of PDGF-BB can significantly improve hDPSCs proliferation, angiogenesis, and odontogenic differentiation, with dentin–pulp complex regeneration in vivo [126]. The latest research direction is the use of exosomes. Zhou et al. proved that exosomes derived from DPSCs of periodontally compromised teeth, possess proangiogenic effects, meaning that they might promote vascularization in regenerative endodontic therapy [129]. He also pointed out that exosomes isolated from cells under an inflammation status, present a much higher proangiogenic potential. Although angiogenesis is the key factor in the dental-pulp regeneration, exosomes also exhibit great potential in cell proliferation, migration and differentiation, suggesting that future research in the use of exosomes in clinical human pulp regeneration is compulsory [123].

### 3.2. Periodontal Disease

Right next to the dental carie, periodontitis is a frequent disease of the periodontium complex, including gingiva, cementum, periodontal ligaments, and alveolar bone, traditionally treated by surgical means and guided tissue regeneration, with limited results [130]. In terms of periodontal regeneration based on dental MSCs, DPSCs, PDLSCs, SCAP, and DFSCs were taken into consideration for several studies [120,131]. For example, local injection of SCAP improved gingival attachment and enhanced bone and cementum regeneration [130]. Another study evaluated the treatment of periodontal intrabony defect with autologous PDLSCs in combination with xenografts (guided tissue regeneration—GTR), proving an increased bone height and no significant adverse effects [132].

Tissue engineering for periodontal tissue regeneration respects the same direction as for the endodontics diseases: stem cells therapy, scaffold supported strategy combined with growth factors, extracellular vesicles (exosomes) with conditioned medium [131,133,134].

One important aspect to be mentioned is that growth factors are released by stem cells and that the PDLSCs conditioned medium improves the periodontal response by suppressing the inflammatory reaction via tumor necrosis factor-alpha (TNF-α) production. Growth factors that promote this type of tissue regeneration are: BMP-2 (bone morphogenetic protein 2), FGF2 (fibroblast growth factor 2), and PDGF (platelet derived growth factor), factors that migration and proliferation of PDL cells [13]. The transplanted conditioned medium (CM) obtained from cultured periodontal ligament stem cells (PDLSCs), decreases mRNA level of TNF-α, therefore, improving healing of periodontal pockets in a study conducted on a rat periodontal defect model [135].

Regarding the use of exosomes, a recent paper published by Liu et al. identifies and suggests the important role in bone regeneration of PDLSC-derived exosomes, naming several differentially expressed exosomal miRNAs, such as miR-122-5p, miR-142-5p, miR-25-3p, miR-192-5p, as positive regulators of osteogenesis [134].

**Figure 2:**
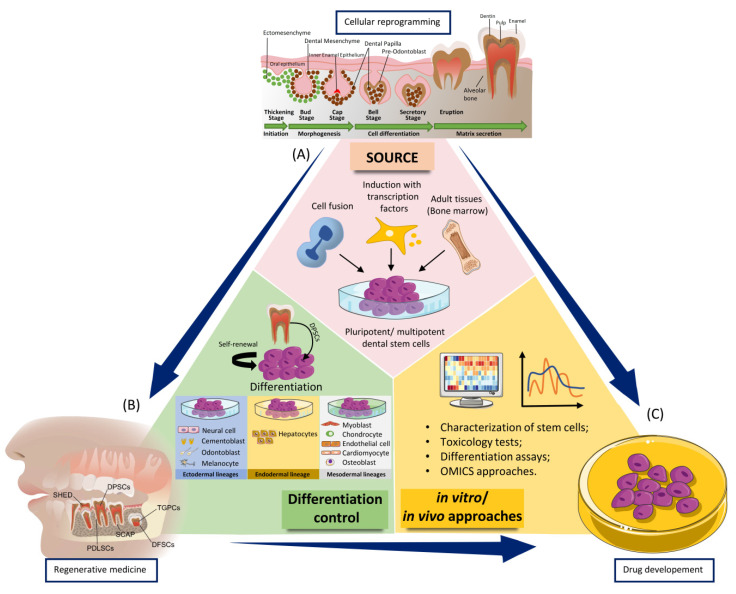
The main applications of the dental stem cells in regenerative medicine, differentiation control, drug design, and testing in dental field. Most applications required three basic steps: (**A**) obtaining pluripotent/multipotent dental stem cells from sources such as cell fusion, induction with transcription factors (Oct3/4, Sox2, c-Myc, Klf4, Oct4, Sox2, Nanog, and Lin28) and from adult tissues such as bone marrow. Pluripotent/multipotent dental stem cells can be used in differentiation as well as in subsequent in vitro/in vivo approaches; (**B**) Dental pulp stem cells (DPSCs) have the ability to self-renewal and differentiation into different cell types of the three germ layers: endoderm, mesoderm, and ectoderm. After the differentiation process, the cells can be used in various in vitro/in vivo approaches; (**C**) in vitro/in vivo approaches to dental stem cells to determine their different functions.

Considering the different differentiation potential of the dental stem cells, these features were exploited in regenerative dentistry for three major directions as resource for cellular reprogramming in regenerative medicine, for control the capacity to induce self-renewal or differentiation into a desired lineage [97,136], of novel testing of novel agents to determine their state or function with important implication on regenerative medicine (Figure 2).

Generally dental stem cells it was observed to sustain the angiogenic potential to a certain degree in vitro, the effect being more complex on the case of the in vivo studies, where the microenvironment at the time of transplantation also promoted the inherent hard tissue-forming potential of the stem cells [137], which potentially diminished the induction of angiogenesis. In clinical practice, dental stem cells did not show a higher vascularization rate in comparison to control constructs, which was perhaps due to odontogenic and/or osteogenic differentiation of the stem cells in the presence/absence of different scaffolds [136].

The scaffold-free approach has been considered a bottom-up strategy that uses different in vitro models as building blocks. This innovative method is based on a cellular reprogramming approach focused on secreting a favorable extracellular matrix and fusing into larger tissue constructs [124]. This can be done only when the process of cellular programming is completely understood, and then to be exploited in regenerative medicine or drug development.

## 4. Conclusions

The oral cavity is of utmost importance in terms of sources of various stem cells. The fact that many of these cells are available during lifetime for many years and that they are easy to harvest, having a low immunogenicity and a great differentiation potential, making them an interesting research topic with high and rapid development. The entire medicine field, including dentistry, has taken a new direction over the past decades in terms of regeneration. Many xenografts have been developed, as well as bone substitutes, different dental implant materials and surfaces. The fact that we now have the tools to harvest and improve the stem cells ability to promote the angiogenic process, by over-expressing certain genes or creating different scaffolds and micro-environments is helping researchers and subsequently practitioners to be more predictive in their maneuvers. Studies need to be conducted in order to establish precise protocols of preservation and manipulation of these unique cells, particular when combining scaffold-based and scaffold-free strategies an optimal solution to circumvent some of the major drawbacks of the current methods while concurrently fostering their advantages.

## Figures and Tables

**Table 1:** Surface markers of MSCs of dental origin

	Expression	Lack of Expression
Minimal criteria of MSCs as defined by ISCT	CD73/5′-Nucleotidase	CD34
	CD90/Thy1	CD45
	CD105/Endoglin	CD11b or CD14
		CD79 alpha or CD19 alpha
		HLA-II
Other common surface markers of dental MSCs	STRO-1	
	SSEA-4	
	CD349	
	3G5	

**Table 2:** Types of stem cells used in regenerative dentistry, their origin, in vitro differentiation potential and therapeutic applications in regenerative dentistry.

MSC	Origin	In Vitro Differentiation	Therapeutic Application in the Dental Field	References
BM-MSCs	Non-hematopoietic components of bone marrow	Osteoblast Adipocyte Chondrocyte Endothelial cells Neural lineage Hepatocytic lineage	Accelerate bone healing and increased bone quantity Angiogenesis by activating VEGF family factors, subsequently improving bone repair process	[44,45,108]
DPSCs	Dental pulp	Osteoblast Odontoblast Adipocyte Neural crest cells	Regenerative endodontics procedures Periodontal regeneration Osteogenic differentiation and subsequently, bone regeneration	[45,57,59,63]
SHED	Pulpal tissue of deciduous teeth	Osteoblast Odontoblast Neural cells Hepatocyte Endothelial cells	Dentin-pulp regeneration Bio-root and periodontal regeneration Accelerated angiogenesis Reduced inflammation and apoptosis Maintain normal salivary workflow (Sjögren syndrome)	[72,76,121]
PDLSCs	Mature periodontal ligament	Osteoblast Adipocyte Cementoblast Fibroblast	Periodontal tissue regeneration Alveolar bone regeneration	[80,81,83]
SCAP	Apical papilla from an immature tooth root	Odontoblast Osteoblast Neurogenic Adipocyte Chondrocyte Hepatocyte	Endodontics Bio-root engineering Periodontal regeneration Bone repair Pro-angiogenic effect	[90,91,92,94,96]
GMDSCs	Gingival connective tissue	Osteoblast Adipocyte Chondrocyte Endothelial cells Neural cells	Blood vessel formation	[95,108]
hPCy-MSCs	Human periapical cyst	osteogenic, adipogenic, and chondrogenic lineage	Regenerative medicine, brain regeneration	[49,51]
DFSCs	Dental follicle	Cementoblast Osteoblast Chondrocyte Adipocyte	Angiogenesis Periodontal/bone regeneration Immunomodulation	[113]

BM-MSC—Bone Marrow Mesenchymal Stem Cells; DPSCs—Human Dental Pulp Stem Cells; SHED—Human Exfoliated Deciduous Teeth; PDLSC—Periodontal Ligament Stem Cells; SCAP—Stem Cells from Apical Papilla; GMDSCs—Gingival-Mesenchymal Derived Stem Cells; hPCy-MSCs—Human Periapical Cyst-Mesenchymal Stem Cells; DFSCs—Dental Follicle Stem Cells.

## Data Availability

Not applicable.
